# Gene–Environment Interaction Affects Risk of Atopic Eczema: Population and In Vitro Studies

**DOI:** 10.1111/all.16605

**Published:** 2025-06-04

**Authors:** Marie Standl, Ashley Budu‐Aggrey, Luke J. Johnston, Martina S. Elias, S. Hasan Arshad, Peter Bager, Veronique Bataille, Helena Blakeway, Klaus Bønnelykke, Dorret Boomsma, Ben M. Brumpton, Mariona Bustamante Pineda, Archie Campbell, John A. Curtin, Anders Eliasen, João P. S. Fadista, Bjarke Feenstra, Trine Gerner, Carolina Medina‐Gomez, Sarah Grosche, Kristine B. Gutzkow, Anne‐Sofie Halling, Caroline Hayward, John Henderson, Esther Herrera‐Luis, John W. Holloway, Joukejan Hottenga, Jonathan O’B Hourihane, Chen Hu, Kristian Hveem, Amaia Irizar, Bénédicte Jacquemin, Leon Jessen, Sara Kress, Ramesh J. Kurukulaaratchy, Susanne Lau, Sabrina Llop, Mari Løset, Ingo Marenholz, Dan Mason, Daniel L. McCartney, Mads Melbye, Erik Melén, Camelia Minica, Clare S. Murray, Tamar Nijsten, Luba M. Pardo, Suzanne Pasmans, Craig E. Pennell, Maria R. Rinnov, Gillian Santorelli, Tamara Schikowski, Darina Sheehan, Angela Simpson, Cilla Söderhäll, Laurent F. Thomas, Jacob P. Thyssen, Maties Torrent, Toos van Beijsterveldt, Alessia Visconti, Judith M. Vonk, Carol A. Wang, Cheng‐Jian Xu, Ali H. Ziyab, UK Translational Research Network in Dermatology, BIOMAP Consortium, Adnan Custovic, Paola Di Meglio, Liesbeth Duijts, Carsten Flohr, Alan D. Irvine, Gerard H. Koppelman, Young‐Ae Lee, Nick J. Reynolds, Catherine Smith, Sinéad M. Langan, Lavinia Paternoster, Sara J. Brown

**Affiliations:** ^1^ Institute of Epidemiology, Helmholtz Zentrum München – German Research Center for Environmental Health Neuherberg Germany; ^2^ German Center for Child and Adolescent Health (DZKJ), partner site Munich Munich Germany; ^3^ MRC Integrative Epidemiology Unit, Bristol Medical School University of Bristol Bristol UK; ^4^ Centre for Genomic and Experimental Medicine, Institute of Genetics and Cancer University of Edinburgh Edinburgh UK; ^5^ Clinical and Experimental Sciences, Faculty of Medicine University of Southampton Southampton UK; ^6^ NIHR Southampton Biomedical Research Centre University Hospitals Southampton NHS Foundation Trust Southampton UK; ^7^ Asthma and Allergy Research Centre Isle of Wight UK; ^8^ Department of Epidemiology Research Statens Serum Institute Copenhagen Denmark; ^9^ Department of Twin Research and Genetic Epidemiology King’s College London London UK; ^10^ COPSAC, Copenhagen Prospective Studies on Asthma in Childhood, Herlev and Gentofte Hospital University of Copenhagen Copenhagen Denmark; ^11^ Department of Biological Psychology Faculty of Behavioral and Movement Sciences, Vrije Universiteit Amsterdam the Netherlands; ^12^ Department of Public Health and Nursing, HUNT Center for Molecular and Clinical Epidemiology Norwegian University of Science and Technology Trondheim Norway; ^13^ ISGlobal, Institute for Global Health Barcelona Spain; ^14^ Universitat Pompeu Fabra (UPF) Barcelona Spain; ^15^ Spanish Consortium for Research on Epidemiology and Public Health (CIBERESP) Madrid Spain; ^16^ Division of Immunology, Immunity to Infection and Respiratory Medicine, School of Biological Sciences The University of Manchester, Manchester Academic Health Science Centre, and Manchester University NHS Foundation Trust Manchester UK; ^17^ Department of Dermatology and Allergy, Herlev and Gentofte Hospital University of Copenhagen Hellerup Denmark; ^18^ Department of Internal Medicine, Erasmus MC University Medical Center Rotterdam Rotterdam the Netherlands; ^19^ Max‐Delbrück‐Center (MDC) for Molecular Medicine Berlin Germany; ^20^ Division of Climate and Environmental Health, Department of Air Quality and Noise Norwegian Institute of Public Health (NIPH) Oslo Norway; ^21^ Department of Dermatology and Venereology, Bispebjerg Hospital University of Copenhagen Copenhagen N Denmark; ^22^ Department of Epidemiology, Bloomberg School of Public Health Johns Hopkins University Baltimore Maryland USA; ^23^ Human Development and Health, Faculty of Medicine University of Southampton Southampton UK; ^24^ Royal College of Surgeons in Ireland Dublin Ireland; ^25^ Children’s Health Ireland Dublin Ireland; ^26^ INFANT Centre University College Cork Cork Ireland; ^27^ The Generation R Study Group Erasmus MC, University Medical Center Rotterdam Rotterdam the Netherlands; ^28^ Department of Dermatology Erasmus MC, University Medical Center Rotterdam Rotterdam the Netherlands; ^29^ Department for Research, St. Olavs Hospital Trondheim University Hospital Trondheim Norway; ^30^ Department of Preventive Medicine and Public Health UPV/EHU Leioa Spain; ^31^ Biogipuzkoa HRI Donostia Spain; ^32^ Univ Rennes, Inserm, EHESP, Irset (Institut de recherche en santé, environnement et travail) – UMR_S 1085 Rennes France; ^33^ IUF‐Leibniz Research Institute for Environmental Medicine Düsseldorf Germany; ^34^ Department of Pediatric Respiratory Medicine, Immunology, and Intensive Care Medicine Charité‐Universitätsmedizin Berlin Berlin Germany; ^35^ Epidemiology and Environmental Health Joint Research Unit FISABIO‐Universitat Jaume I–Universitat de València Valencia Spain; ^36^ Department of Dermatology, Clinic of Orthopedy, Rheumatology and Dermatology St. Olavs Hospital, Trondheim University Hospital Trondheim Norway; ^37^ Clinic for Pediatric Allergy, Experimental and Clinical Research Center Charité University Medical Center Berlin Germany; ^38^ Bradford Institute for Health Research Bradford Teaching Hospitals NHS Foundation Trust Bradford UK; ^39^ Danish Cancer Institute Copenhagen Denmark; ^40^ Department of Clinical Medicine University of Copenhagen Copenhagen Denmark; ^41^ Department of Pediatrics Stanford University School of Medicine Stanford California USA; ^42^ Department of Clinical Science and Education, Södersjukhuset Karolinska Institutet Stockholm Sweden; ^43^ School of Medicine and Public Health The University of Newcastle Newcastle New South Wales Australia; ^44^ Mothers and Babies Research Program Hunter Medical Research Institute Newcastle New South Wales Australia; ^45^ Department of Neonatology, Rigshospitalet University of Copenhagen Copenhagen Denmark; ^46^ University College Cork Cork Ireland; ^47^ Cork University Hospital Cork Ireland; ^48^ Department of Women’s and Children’s Health Karolinska Institutet Stockholm Sweden; ^49^ Astrid Lindgren’s Children’s Hospital Karolinska University Hospital Stockholm Sweden; ^50^ Department of Clinical and Molecular Medicine Norwegian University of Science and Technology Trondheim Norway; ^51^ BioCore – Bioinformatics Core Facility Norwegian University of Science and Technology Trondheim Norway; ^52^ Area de Salut de Menorca, ib‐salut Balearic Islands Spain; ^53^ Department of Clinical and Biological Sciences, Center for Biostatistics, Epidemiology and Public Health University of Turin Turin Italy; ^54^ Department of Epidemiology University of Groningen, University Medical Center Groningen Groningen the Netherlands; ^55^ University of Groningen, University Medical Center Groningen, Groningen Research Institute for Asthma and COPD (GRIAC) Groningen the Netherlands; ^56^ Centre for Individualised Infection Medicine (CiiM), a joint venture between the Helmholtz Centre for Infection Research (HZI) and Hannover Medical School (MHH) Hannover Germany; ^57^ TWINCORE, a joint venture between the Helmholtz‐Centre for Infection Research (HZI) and the Hannover Medical School (MHH) Hannover Germany; ^58^ Department of Community Medicine and Behavioral Sciences, College of Medicine Kuwait University Kuwait City Kuwait; ^59^ National Heart and Lung Institute Imperial College London London UK; ^60^ St John’s Institute of Dermatology and the School of Basic and Medical Sciences King’s College London London UK; ^61^ Division of Respiratory Medicine and Allergology, and Neonatology, Department of Pediatrics Erasmus MC, University Medical Center Rotterdam Rotterdam the Netherlands; ^62^ Division of Neonatology, Department of Neonatal and Intensive Care Erasmus MC, University Medical Center Rotterdam Rotterdam the Netherlands; ^63^ Department of Paediatric Dermatology, St John’s Institute of Dermatology Guy’s and St Thomas’ NHS Foundation Trust and King’s College London London UK; ^64^ Department of Clinical Medicine Trinity College Dublin Dublin Ireland; ^65^ Department of Pediatric Pulmonology and Pediatric Allergology, Beatrix Children’s Hospital University of Groningen, University Medical Center Groningen Groningen the Netherlands; ^66^ Faculty of Medical Sciences, Translational and Clinical Research Institute Newcastle University Newcastle upon Tyne UK; ^67^ Department of Dermatology and NIHR Newcastle Biomedical Research Centre Newcastle upon Tyne Hospitals NHS Foundation Trust Newcastle upon Tyne UK; ^68^ Faculty of Epidemiology and Population Health London School of Hygiene & Tropical Medicine London UK; ^69^ St John’s Institute of Dermatology Guy’s and St Thomas’ NHS Foundation Trust London UK; ^70^ NIHR Bristol Biomedical Research Centre University Hospital Bristol and Weston NHS Foundation Trust and University of Bristol Bristol UK; ^71^ Department of Dermatology NHS Lothian Edinburgh, Scotland UK

**Keywords:** atopic eczema, dog, environment, gene, interaction

## Abstract

**Background:**

Multiple environmental and genetic factors play a role in the pathogenesis of atopic eczema (AE). We aimed to investigate gene–environment interactions (G × E) to improve understanding of the pathophysiology.

**Methods:**

We analysed data from 16 European studies to test for interaction between the 24 most significant AE‐associated loci identified from genome‐wide association studies and 18 early‐life environmental factors. We tested for replication using a further 10 studies and in vitro modeling to independently assess findings.

**Results:**

The discovery analysis (including 25,339 individuals) showed suggestive evidence for interaction (*p* < 0.05) between seven environmental factors (antibiotic use, cat ownership, dog ownership, breastfeeding, elder sibling, smoking and washing practices) and at least one established variant for AE, 14 interactions in total. In the replication analysis (254,532 individuals) dog exposure × rs10214237 (on chromosome 5p13.2 near *IL7R*) was nominally significant (OR_interaction_ = 0.91 [0.83–0.99] *p* = 0.025), with a risk effect of the T allele observed only in those not exposed to dogs. A similar interaction with rs10214237 was observed for siblings in the discovery analysis (OR_interaction_ = 0.84 [0.75–0.94] *p* = 0.003), but replication analysis was under‐powered (OR_interaction_ = 1.09 [0.82–1.46]). rs10214237 homozygous risk genotype is associated with lower IL‐7R expression in human keratinocytes, and dog exposure modelled in vitro showed a differential response according to rs10214237 genotype.

**Conclusion:**

Interaction analysis and functional assessment provide preliminary evidence that early‐life dog exposure may modify the genetic effect of rs10214237 on AE via *IL7R*, supporting observational epidemiology showing a protective effect for dog ownership. The lack of evidence for other G × E studied here implies only weak effects are likely to occur.

## Graphical abstract

Atopic eczema is a complex trait resulting from multiple genetic and environmental factors. This study investigated evidence for interaction between 24 established eczema genetic risk variants and 18 environmental factors. One variant‐environmental pair showed nominally significant interaction in discovery, replication and meta‐analyses: early‐life dog exposure modulates the increased risk of rs10214237 and preliminary work suggests a mechanistic effect via IL‐7R. CCL2, C‐C motif chemokine ligand 2; chr 5, chromosome 5; CSF2, colony stimulating factor 2 (granulocyte‐macrophage); CXCL8, C‐X‐C motif chemokine ligand 8 (IL‐8); ELISA, enzyme‐linked immunosorbent assay; FLG, filaggrin; GWAS, genome‐wide association study; IL, interleukin; *n*, number of participants; OR, odds ratio; RT‐qPCR, reverse transcription quantitative polymerase chain reaction; TNF, tumour necrosis factor; TSLP, thymic stromal lymphopoietin.

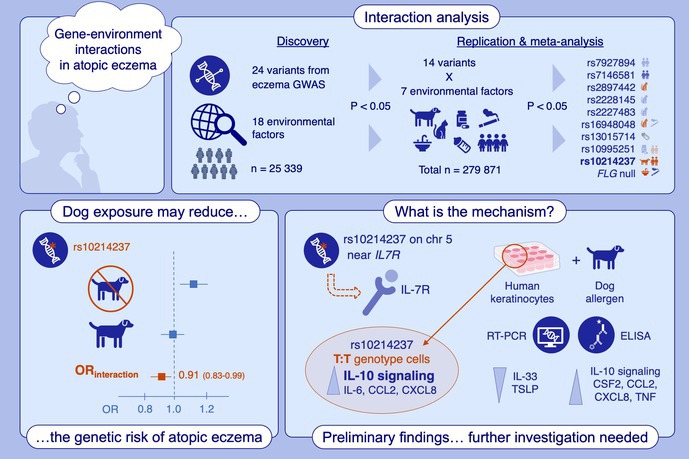

## 1 Background

Atopic eczema (AE, synonymous with atopic dermatitis or eczema [1]) is a chronic inflammatory skin and systemic condition affecting approximately 20% of children and 10% of adults in high‐income countries. Eczema is the dermatosis that contributes the greatest number of disability‐adjusted life years worldwide [2] and co‐morbid conditions, including asthma and allergies, obesity, cardiovascular disease, anxiety and depression, add substantially to the social, academic, occupational and financial impact [3]. Atopic eczema is a heritable trait [4] but the rapid rise in prevalence in industrialised areas over the past 30 years [3, 5] illustrates the importance of environmental factors in aetiology. A greater understanding of environmental effects in driving pathology could facilitate disease prevention.

The European Academy of Allergy and Clinical Immunology published an umbrella review of systematic reviews and identified a lack of research in eczema genetic epidemiology and environmental effects [6]. The investigation of environmental factors using observational epidemiology is inherently challenging in the context of AE because of multiple confounding factors and possible reverse causation [7]. Genetic studies, however, have made substantial progress in defining mechanisms in eczema predisposition and pathogenesis, including skin barrier dysfunction and aberrant immune response [8]. The evidence of individual variation in susceptibility to environmental allergens and irritants supports the concept of gene–environment interaction (G × E) [9] playing a role in AE. Loss‐of‐function variants in *FLG*, encoding the skin barrier protein filaggrin, have been implicated [10] but studies lack statistical power. Knowledge of genetic risk may provide an opportunity to identify key environmental effects and clarify important disease biology.

We aimed to investigate evidence of interaction between the most highly significant AE risk loci defined by genome‐wide association studies [11] and environmental risk factors defined by previous literature [7, 10] and of importance to patients and carers [12]. We used early‐life environmental exposures (in utero and up to the first 24 months of life) to minimize reverse causation. G × E was tested in large cohorts from European populations, in discovery and replication phases, and mechanistic assessment was carried out in vitro using a skin keratinocyte model to validate interactions.

## 2 Methods

### 2.1 Genetic and Environmental Factors

Genetic risk loci were defined by the 24 top hits at each locus identified in European‐ancestry AE genome‐wide association studies [11, 13] and coded for the risk‐increasing allele as effect allele (File S1). *FLG* null genotype was coded as presence/absence (0/1) of any of the loss‐of‐function variants prevalent in the white European population (R501X, 2282del4, R2447X, S3247X as previously reported [11, 14]).

Environmental exposures in utero or up to 24 months of age were selected on the basis of our literature review [10] and in discussion with representatives from a national eczema support group [12]. Data was available to allow analysis of 18 environmental effects: pet ownership for cat and dog separately; house dust mite exposure (at birth or 1 year); washing practices (at 6 months or 2 years); cigarette smoking within the household (in utero or up to 2 years); antibiotic use (6 or 12 months); environmental pollution (PM10 or at 5 months); breast feeding (ever and duration); mode of delivery; and presence of older siblings. These are listed in Files S2 and S3, with details of their definition and coding.

G × E effects showing a nominally significant interaction in the discovery and replication analyses were taken forward for in vitro modeling.

### 2.2 Population Cohorts

Sixteen population‐based cohorts from people of European ancestry (*N* = 25,339) were included in the discovery analysis and a further 10 European population‐based cohorts in the replication stage (*N* = 254,532), giving a maximum total of 279,871 (max*N*) in the final meta‐analysis (File S4). AE was defined by parental report or doctor diagnosis for those who had ‘ever had eczema’. Further details on the phenotype definitions can be found in File S2.

Each cohort has ethical approval for the sharing of anonymised data from study participants, with their written informed consent.

### 2.3 Statistical Genetic Analysis

To test for G × E, a statistical model was fitted to include the main effect of each genetic variant upon eczema (G) (extracted from Paternoster et al. [11]), the main effect of the environmental factor upon eczema (E), and the product of the genetic effect and the environmental effect (G × E). Logistic regression models were applied to identify the main effect of each environmental factor (models M1–M4, File S5), and to test for interaction between the exposure and each SNP while adjusting for sex (models I1–I3, File S5). Sensitivity analyses were performed adjusting for family history of atopic disease (asthma, eczema or hay fever) and parental education as a proxy for socioeconomic status (models S1–S3, File S5).

Analyses were conducted separately within each cohort and then combined by fixed‐effects meta‐analysis. Genetic data was imputed separately for each cohort, detailed in File S2.

### 2.4 Power Calculation

Posthoc estimates of statistical power were calculated in Quanto (version 1.2.4). These were informed by effect size estimates from the discovery analyses or previously published studies, assuming a case‐to‐control ratio of 1:3 and *α* = 0.004 in replication analyses (0.05/14 for multiple testing of 14 gene–environment pairs) (File S6).

### 2.5 Review of In Silico Data for Genetic Variants

Variants showing statistical evidence of G × E were investigated using available data from previous publications [11, 13], the NHGRI‐EBI GWAS Catalogue (https://www.ebi.ac.uk/gwas/), LDLink (version 5.6.6), the Genome Aggregation Database (gnomAD v4.1.0, https://gnomad.broadinstitute.org/), UCSC Genome Browser (https://genome.ucsc.edu/), Open Targets (https://www.opentargets.org/), GTEx Portal (https://www.gtexportal.org/home/) and the Human Protein Atlas [15, 16].

### 2.6 Keratinocyte Culture and Gene Expression

Full details are provided in File S7. In brief, primary human keratinocytes were isolated from normal human skin samples and genotyped for rs10214237 using KASP (LGC Genomics, Teddington, England). *IL7R* mRNA expression was quantified in 34 keratinocyte samples (3 of C:C genotype, 15 T:C and 16 T:T) using RT‐qPCR. Fold changes in gene expression were derived via the 2(−Delta Delta C[T]) method, using *EF1A* as the reference gene.

### 2.7 In Vitro Analysis for rs10214237 × Dog Interaction

Human keratinocytes comprise the outermost layer of skin and can therefore represent the first line of interaction in an allergen encounter in utero or early life. To further investigate the effect of dog exposure in early life, primary normal human keratinocytes were exposed to clinical‐grade dog epithelial extract, a standardized reagent used for allergy testing in the clinic [17]. Methods are described in full in File S7. Briefly, gene expression was quantified using RT‐qPCR, and cytokine, chemokine, and receptor expression was quantified using an ELISA array. Experiments were replicated using keratinocytes from a minimum of five independent donors. Gene ontology, network and pathway analyses were conducted using STRING v12.0.

## 3 Results

Our analysis investigated the interaction between 24 variants identified in European GWAS studies [11, 13] and 18 environmental exposures selected through literature review (described in Section 2). We first assessed the observational association (of environmental effects) followed by testing for interaction effect (of environmental and genetic risk factors) in the discovery cohorts; next, the nominally significant findings and those with a priori evidence were tested for replication in available larger cohorts.

### 3.1 Discovery Analysis

Meta‐analyses of between 1084 and 22,263 participants (numbers dependent on exposure, File S4) showed strong evidence for antibiotic use increasing the risk of AE (in utero *p* = 0.004, at 6 months *p* = 0.001 and at 12 months *p* = 6 × 10^−4^); weaker evidence was found for a protective effect of dog ownership (*p* = 0.03), a protective effect of childhood smoke exposure (*p* = 0.038) and a risk effect of NO_2_ levels (*p* = 0.035) (M1 models, File S8). Little or no evidence (*p* > 0.05) was found for effects of caesarean delivery, cat ownership, breastfeeding, elder siblings, in utero smoke exposure, washing practices at 6 months and 2 years, PM10 exposure, and house dust mite exposure at birth or 1 year (M1 models, File S8).

**FIGURE 1:**
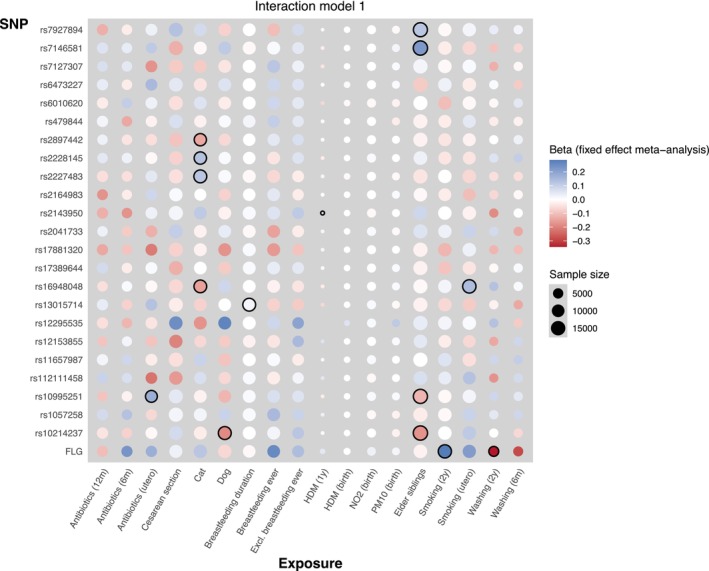
Heatmap to summarise results of interaction analyses. Strength of colour indicates beta in which blue is positive and red is a negative direction of effect; diameter of circle indicates sample size; 14 nominally significant interactions (*p*_int_ < 0.05) are highlighted with black outline; one association was reported in only one cohort, so it was not pursued further.

Of the 432 interactions tested (between 24 genetic variants and 18 environmental exposures), we found 14 nominally significant (*p*_int_ < 0.05) interactions, but no interactions passed multiple testing correction (Figure 1; Table 1). Of the nominally significant interactions, eight indicated a higher genetic risk in the presence of the exposure (OR > 1) and 6 indicated a higher genetic risk in the unexposed stratum (OR < 1). Of the 18 environmental exposures tested, the two with the strongest evidence for interaction with *FLG* null variants were exposure to tobacco smoke between 0 and 2 years (*p*_int_ = 0.018) and washing practices during the same period (*p*_int_ = 0.045). There was little evidence (*p* > 0.05) for interactions between *FLG* null variants and other tested exposures, though confidence intervals for some interaction estimates were wide (File S8). Notably, there was little evidence for interaction between *FLG* null variants and cat exposure (*p* = 0.36), with strong effects of *FLG* in both the unexposed and exposed strata.

**TABLE 1:** Nominally significant interaction results from discovery and replication analyses.

Exposure	SNV or gene	Discovery							Replication					*p* combined
		*N*	*N* studies	OR	[95% CI]	*p*	*p* random	*p* heterogeneity	*N*	*N* studies	OR	[95% CI]	*p*	
Antibiotic use in utero	rs10995251	11,575	7	1.19	[1.00–1.42]	0.045	0.059	0.34	2666	1	0.88	[0.55–1.41]	0.59	0.09
Cat ownership	rs16948048	14,063	10	0.86	[0.76–0.97]	0.012	0.29	0.054	49,212	3	1	[0.92–1.08]	0.98	0.17
Cat ownership	rs2227483	14,644	11	1.13	[1.01–1.27]	0.037	0.18	0.014	49,212	3	1.05	[0.97–1.13]	0.25	0.036
Cat ownership	rs2228145	12,994	9	1.14	[1.01–1.29]	0.037	0.037	0.61	49,212	3	1	[0.92–1.08]	0.93	0.3
Cat ownership	rs2897442	12,702	9	0.87	[0.75–1.00]	0.044	0.11	0.35	49,212	3	1.02	[0.94–1.11]	0.59	0.58
Dog ownership	rs10214237	14,656	11	0.83	[0.72–0.96]	0.011	0.014	0.37	47,185	2	0.91	[0.83–0.99]	0.025	0.0013
Duration of any breastfeeding	rs13015714	14,474	9	1.02	[1.00–1.03]	0.049	0.068	0.32	4252	5	0.99	[0.95–1.02]	0.47	0.14
Elder siblings	rs10214237	19,155	11	0.84	[0.75–0.94]	0.003	0.003	0.99	5049	4	1.09	[0.82–1.46]	0.55	0.011
Elder siblings	rs10995251	18,608	10	0.89	[0.80–1.00]	0.042	0.042	0.44	7529	6	1.03	[0.85–1.25]	0.74	0.11
Elder siblings	rs7146581	19,176	11	1.25	[1.10–1.42]	0.00042	0.0031	0.32	7529	6	1.04	[0.84–1.29]	0.73	0.0012
Elder siblings	rs7927894	18,263	10	1.13	[1.01–1.26]	0.031	0.031	0.53	7529	6	1	[0.83–1.21]	0.97	0.058
Smoking in household up to 2 years	*FLG* ^a^	15,618	12	1.33	[1.05–1.68]	0.018	0.022	0.36	147,880	7	1.01	[0.81–1.25]	0.93	0.096
Smoking in household in utero	rs16948048	16,062	11	1.15	[1.02–1.31]	0.028	0.028	0.53	8078	5	1.04	[0.85–1.27]	0.72	0.039
Washing practices up to 2 years	*FLG* ^a^	6962	3	0.71	[0.51–0.99]	0.045	0.12	0.31	1061	1	1.93	[0.29–12.99]	0.5	0.063

*Note:* Results of testing for interaction between 24 genetic variants and 18 environmental exposures (File S3). Nominal significance defined as unadjusted *p*_int_ < 0.05.

Abbreviations: *N*, number; OR, odds ratio; *p*, significance from fixed effects meta‐analysis; *p* combined, significance from combined fixed effects meta‐analysis of discovery and replication data*p* random, significance from random effects meta‐analysis.

^a^ Combined null genotype for two or more loss‐of‐function mutations in *FLG* as detailed in cohort descriptions (File S2).

Sensitivity analyses, additionally adjusting for family history of AE and socioeconomic status, supported the main analyses (File S8), but many of the sensitivity analyses were based on much smaller sample sizes because of the requirement for data on additional covariates. There was little evidence of heterogeneity between cohorts (smallest *p*_het_ = 0.01) amongst the 14 reported interactions.

### 3.2 Replication Analysis

Fourteen G × E interactions with nominal evidence were tested for replication. Additionally, exposures previously reported to interact with *FLG* null variants (cat, siblings and breast‐feeding [10]) were included in the replication analysis, making a total of 19 G × E interactions (8 exposures and 10 genetic variants) (File S9).

**FIGURE 2:**
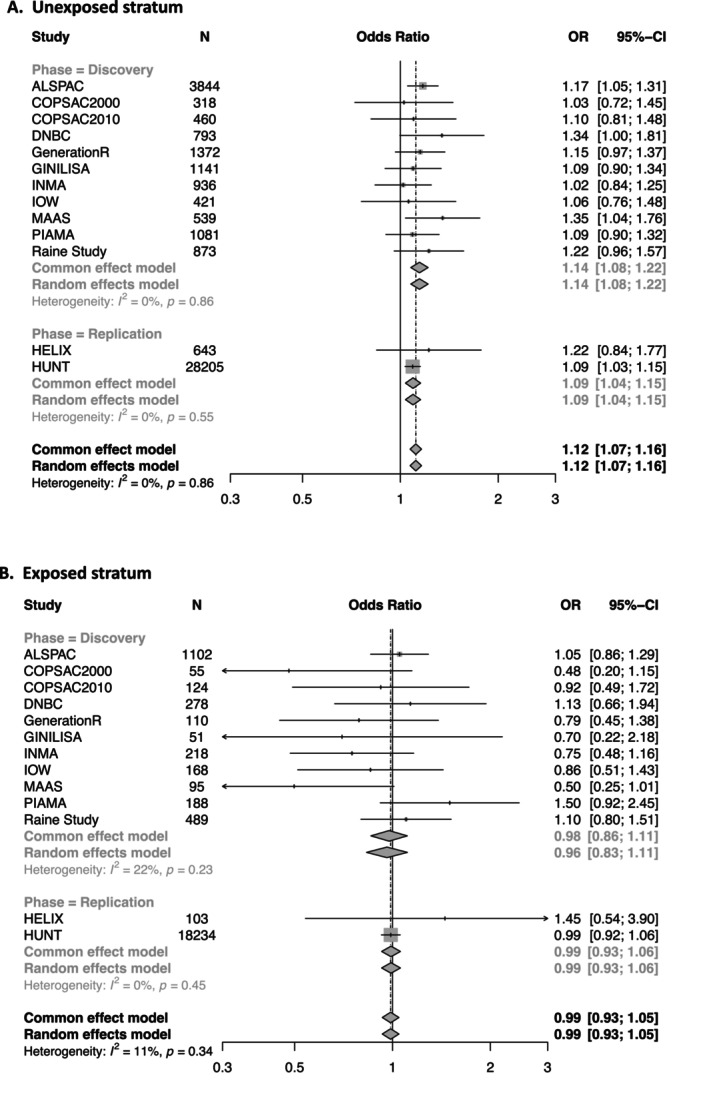
Forest plot showing interaction of dog exposure with rs1041237 in (A) unexposed and (B) exposed strata. Interaction analysis for discovery (*N* = 18,045), replication (*N* = 47,185) and combined meta‐analysis (total *N* = 65,230) show the T allele of rs1041237 increases the risk of atopic eczema only amongst those who are not exposed to a dog in the family home. Full names and study cohort descriptions are given in File S2.

Dog exposure and rs1041237 showed evidence for interaction (*p*_int_ = 0.025, Table 1). In an analysis stratified by dog exposure, the T allele increases the risk of atopic eczema (OR = 1.14, 95% CI 1.08–1.22), but only amongst those who are not exposed to a dog in the family home. In individuals who are exposed to a dog in early life, this variant appears to have little or no effect (OR = 0.99, 95% CI 0.93–1.05, Figure 2).

Availability of environmental data for replication varied, with many of our attempted replications of interactions being insufficiently powered to be conclusive. Washing practices (0–2 years) and antibiotic use in utero interactions had only 3% and 4% power, respectively (File S6). The tobacco exposure in utero interaction had only 11% power, and the sibling interactions had 8%–37% power (dependent on variant). The breast‐feeding duration interaction had 4% power in the replication phase, and so we extended the replication analysis to ‘ever breastfed’ to increase the power to 56%. The interactions with dog, cat and tobacco smoke exposure 0–2 years were all sufficiently powered (88%, 72%–88% and 99%, respectively). The previously reported interactions between *FLG* null mutations and cat, siblings, and ever breastfed had 99% power, based on their previously reported interaction effects (File S6).

### 3.3 In Silico Follow‐Up of rs10214237

rs10214237 is an intergenic variant (T>C) on chromosome 5p13.2; an association with AE was identified by genome‐wide association study (GWAS) [11] in which *IL7R* was prioritized as the likely causal gene based on evidence including eQTL colocalization in macrophages and monocytes [13, 18]. The top single nucleotide variant (SNV) at this locus in a more recent GWAS meta‐analysis [13] is rs10214273, but this variant is in complete linkage disequilibrium with rs10214237 in European populations (*R* ^2^ = 1, LDLink version 5.6.6, LDPair tool). Global population data from gnomAD shows ancestral differences in allele frequency, with rs10214237 being more frequent in European and South Asian populations (MAF 0.28 and 0.20, respectively) compared to African people (MAF 0.07) (1KG data accessed 10 Jan 2025).

rs10214237 is within a region of open chromatin in keratinocytes and fibroblasts, but not the lymphoblastoid cell line GM12878 (UCSC Genome Browser 6 Feb and 27 Nov 2024). Open Targets V2G analyses confirm *IL7R* as the most likely gene affected by this SNV based on pQTL, sQTL and eQTL (6 Feb and 27 Nov 2024). GTEx data show that the expression of *IL7R* is higher with T:T genotype in whole blood and cultured fibroblasts, and in our newly generated data, we show that individuals with the T:T genotype have slightly higher *IL7R* mRNA expression in primary human keratinocytes than those with the C:C genotype (File S10). Single cell data from the Human Protein Atlas [15, 16] confirm that IL‐7R is expressed at the protein level in human keratinocytes, in addition to circulating immune cells.

### 3.4 In Vitro Testing of the Effects of Dog Allergen on Human Keratinocytes

**FIGURE 3:**
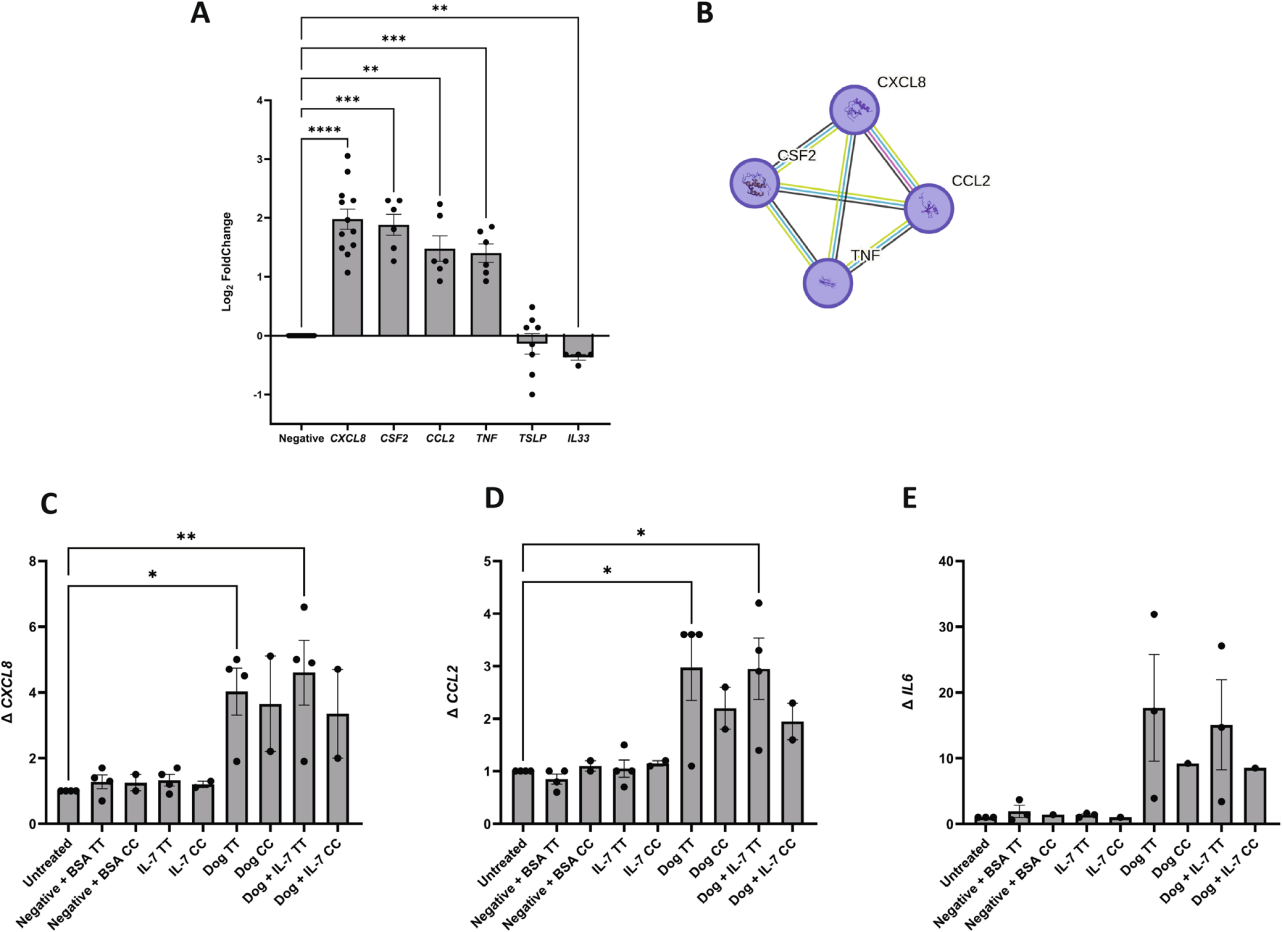
In vitro testing of the effects of dog allergen on primary human keratinocytes. (A) Dog allergen exposure stimulated a reduction in *IL33* and *TSLP* mRNA but upregulation of *CXCL8* (IL‐8), *CSF2*, *CCL2*, and *TNF*; negative indicates keratinocyte media with dog allergen carrier solution; 5–12 donor isolates shown, bars represent SEM one‐way ANOVA, Dunnett post hoc test compared to negative control, **p < 0.01, ***p < 0.001, ****p < 0.0001. (B) IL‐10 signaling was the most significantly enriched Reactome pathway (4 out of 45 genes/proteins, FDR 7.71e‐08). (C–E) Effects of IL‐7 and dog allergen stimulation on primary human keratinocytes with different rs10214237 genotypes in which T is the eczema risk allele; graphs represent the mean fold change in cytokine mRNA expression relative to the housekeeping gene *EF1A*, from four keratinocyte isolates with T:T genotype and two keratinocyte isolates from donors of C:C genotype; untreated indicates keratinocyte media only and negative is keratinocyte media with dog allergen carrier solution; BSA as 0.0002% included for as carrier protein for recombinant Il‐7; two‐way ANOVA with Dunnett's post hoc test, compared to the negative control, bars represent SEM, *p < 0.05, **p < 0.01.

Dog allergen exposure stimulated an up‐regulation in *CXCL8* (IL‐8), *CSF2*, *CCL2* and *TNF* mRNA, but the atopy‐related cytokines *IL33* and *TSLP* mRNA were down‐regulated (Figure 3A). Network analysis of the proteins encoded by the upregulated transcripts showed significant enrichment for IL‐10 signalling (Figure 3B, Reactome pathway FDR 7.71e‐08) which plays a suppressive role in contact dermatitis and atopic eczema [19]. To test the keratinocyte response more broadly, we used an ELISA panel of 64 cytokines, chemokines and receptors (File S11). This confirmed the signature of increased IL‐10 signalling (File S11).

Next, using primary human keratinocytes of known rs10214237 genotype and focusing on CXCL8 (IL‐8), CCL2 and IL‐6 as molecules of relevance to IL‐7R signalling in epithelial cells, we investigated the effect of dog allergen exposure, with and without IL‐7 stimulation (Figure 3C–E). There was no difference in expression levels after IL‐7 stimulation, but on stimulation with dog extract (or IL‐7 plus dog extract), keratinocytes of the T:T genotype (homozygous for the eczema‐risk allele) showed a greater response than the C:C genotype.

This work is preliminary and requires further mechanistic investigation in a larger number of samples, but these observations provide a possible mechanistic explanation for the finding that the T allele at rs10214237 increases risk for atopic eczema. The T:T genotype shows greater IL‐7R mRNA expression, but in the context of dog exposure, the risk effect is overshadowed by an increase in cytokines and chemokines in the IL‐10 pathway that suppress eczema to a greater extent in T:T than C:C individuals.

## 4 Discussion

### 4.1 Large, Systematic Study of G × E in Atopic Eczema

Our collaborative work represents the largest and most comprehensive analysis to date investigating G × E in AE, using a systematic approach focused on the most significant genetic loci and selected environmental factors. We first meta‐analyzed data from available observational studies to test for association and then applied interaction analysis to investigate G × E. Statistical power remains a limiting factor and the nominal significance level (*p* < 0.05 without correction for multiple testing) means cautious interpretation is needed. We have identified important negative results as well as one interaction with functional validation in vitro and others that warrant further follow‐up.

A variety of sources provide evidence that G × E plays a role in the aetiology of AE. These include rapidly rising prevalence [5], clinical observation [4], epidemiological studies [10], and in vitro analyses demonstrating molecular effects that include aryl hydrocarbon receptor signalling [20]. Some authors have even stated that ‘atopic eczema is an environmental disease’ [21]. Our meta‐analysis of observational associations provides evidence that early‐life exposure to antibiotics and NO_2_ levels associates with an increased risk of AE, whilst early‐life exposure to dog or tobacco smoke is associated with a lower risk of AE in the populations studied. However, these associations may be affected by bias through confounding and reverse causation.

### 4.2 Genotype‐Specific Effects of Early‐Life Dog Exposure

Statistical interaction analysis indicates that early‐life dog exposure may modify the genetic effect of rs10214237. Functional genetic analyses show an effect mediated via the gene *IL7R*, which encodes the alpha‐subunit of the IL‐7 receptor. Rs10214237 T:T genotype was associated with an increased risk of atopic eczema in the population as a whole and in the sub‐population without dog exposure (Figure 2) consistent with the T:T genotype showing slightly greater IL7R mRNA expression (File S10). The IL‐7 receptor is a heterodimer composed of IL7R‐alpha and IL2R‐gamma. It is expressed in multiple cell types and tissues, including T‐cells, NK‐cells, glandular, and stratified epithelial cells (data from Human Protein Atlas [15, 16]). IL7R‐alpha also contributes to a heteromeric complex with the thymic stromal lymphopoietin (TSLP) receptor, but our experimental work to test TSLP as an alternative ligand in keratinocytes was not informative (data not shown) likely in part because the TSLPR is only very lowly expressed in keratinocytes [16, 22].

Our detailed work in vitro focused on human epidermal keratinocytes as the earliest tissue to encounter dog allergen in the initiation of atopic disease, in utero or early infancy. We have shown evidence that keratinocytes display a direct response to dog allergen exposure, with down‐regulation of IL‐33 and TSLP mRNA (both inducers of type 2 immune responses in atopy [23, 24]) and upregulation of a network of genes encoding chemokines and cytokines of IL‐10 signaling (Reactome pathway HAS‐6783783), contributing to the suppression of atopic inflammation [19]. This is consistent with observational epidemiology showing an apparent protective effect of dog exposure early in life [25, 26]. Gene ontology analysis of the same network indicates a role in cellular response to lipopolysaccharide (GO:0071222), likely to reflect a response to gram‐negative components of the canine microbiome.

The proposed interaction with genotype was investigated using keratinocytes of known rs10214237 genotype. This is preliminary mechanistic work and further, more detailed investigations are required. However, our analyses indicate a trend in which the T:T cells show a greater increase in IL‐10 signaling in response to dog allergen exposure than C:C. This is consistent with the suppression of atopic eczema risk on a population level in the dog‐exposed T:T individuals, while non‐dog‐exposed T:T individuals remain at risk of disease. The precise mechanistic pathways remain to be defined, but the interaction between rs10214237 and dog exposure in AE risk is analogous to the 17q21 × dog interaction previously demonstrated in asthma, in which the risk of persistent wheeze is attenuated by dog ownership [27]. There is also an interesting parallel in the interaction of rs10214237 with exposure to older siblings, in which the older sibling abrogates the risk effect for rs10214237. We speculate that this may be related to the increased microbial exposure experienced by an infant with older siblings (or a dog) in the household. There is evidence of shared skin and gut microbiomes between humans and their pets [28], but it could also reflect lifestyle choices of dog‐owning families and these hypotheses require further testing.

### 4.3 Lack of Evidence for Previously Reported FLG × Environment Effects

In our previous systematic review focusing on gene–environment interactions with *FLG* null mutations [10] we found some published evidence for *FLG* × environment interactions with exposures including early‐life cat ownership, older siblings, water hardness, phthalate exposure and prolonged breastfeeding from the small number of previous studies. The lack of replication of the *FLG* × cat ownership interaction in the large, well‐powered study reported here and another recent meta‐analysis [29] represents an important null finding. Two small birth cohort studies [30, 31] (*n* = 379 and *n* = 503) reported *p* values for interaction < 0.01 with evidence for increased risks of AE in those with *FLG* null mutations exposed to cat in early life, but the evidence for these G × E interactions came from small numbers of individuals (*n* = 5 in one study [30]) with *FLG* mutation, cat exposure, and development of AE. We had very good power (99%) for the interaction magnitude previously reported (OR_int_ = 11) [30] and 80% for an interaction as small as OR_int_ = 1.26, suggesting very little evidence in our data for *FLG* × cat interaction. We found little evidence for *FLG* × breastfeeding, consistent with our systematic review [10], where studies reported no evidence for interactions with breastfeeding, although an *FLG* × breastfeeding duration interaction was reported from the Isle of Wight cohort [32]. Here, our post hoc power calculation (File S6) showed adequate power (99%) for the *FLG* × breastfed‐ever interaction, but low power (< 1%) for *FLG* × breastfeeding duration analyses, which may explain the discrepancy.

### 4.4 Limitations

The discovery analysis used selected SNVs to represent known eczema risk loci, rather than conducting a genome‐wide interaction analysis. This restricted approach is needed because of power constraints even in large population datasets, and it has been shown to be effective in other traits [33]. A post hoc estimation of statistical power (File S6) showed that our replication sample sizes were insufficient for some interactions. Therefore, where replication results do not meet our pre‐specified significance threshold, it is not possible to definitively *exclude* an interaction, but we report the interaction effect sizes for which we had good statistical power, to demonstrate the magnitude of interactions that are unlikely to exist, given our null results (File S6). Furthermore, by focusing on selected SNVs within the known AE risk loci, we acknowledge that there may be loci in which an effect is only apparent in the context of interaction with an environmental exposure. These would not be detected by our analysis strategy, and genome‐wide interaction analysis should be considered in future work if far larger sample sizes than those used here become available. The prospective collection of detailed environmental data is challenging, and the availability of relevant information has limited our analyses. A specific example is the recording of washing practices, quantified by frequency of bathing but lacking important details such as the type of wash product used.

An important limitation to this work is the use of European cohort data including people of predominantly white ancestry; this reflects the current sparsity of diverse ancestries in population genetic studies of sufficient size to carry out these analyses. The observed differences in allele frequency of rs10214237 in African compared to European and South Asian populations illustrate the limited transferability of this variant effect across populations, although other population‐specific variants in the same locus may contribute to similar mechanistic effects. International efforts are on‐going to address this limitation [34], and future G × E studies are needed to investigate population‐specific environmental effects. More detailed sub‐phenotyping of AE may, in the future, reveal that more specific genetic and environmental drivers exist in distinct ancestral or sub‐phenotype groups.

### 4.5 Conclusions

We report observational evidence for an association of AE with exposure to antibiotics, NO_2_, and tobacco smoke in early life, but the precise nature and mechanisms of action of these environmental factors on atopic skin inflammation remain unclear. We detected an observational association between early life dog exposure and reduction in prevalence of AE, in keeping with previous reports. Further interaction analysis and functional assessments provide evidence that dog exposure reduces the genetic risk effect of rs10214237 in a pathway via *IL7R* and possibly IL‐10, to suppress skin inflammation. There may be an equivalent interaction effect with siblings, but this is not possible to model in vitro. The lack of statistical evidence for other G × E explored in this analysis suggests that only weak interactions are likely to exist, indicating that on a population level the interactions tested and found to be null are unlikely to have important contributions to AE pathogenesis. Future, larger longitudinal studies should therefore focus on alternative mechanistic investigations.

## Author Contributions

A.B.‐A., P.B., K.B., D.B., S.J.B., M.B.P., A.Cu., C.F., J.He., J.W.H., J.O'B.H., A.D.I., G.H.K., P.D.M., S.M.L., Y.‐A.L., D.M., E.M., C.S.M., DMM, S.P., L.P., CP, N.J.R., A.S., C.S., M.S., J.P.T., and C.A.W. made substantial contributions to the conception or design of the work; all authors contributed to the acquisition, analysis, or interpretation of data; A.B.‐A., S.J.B., A.Cu., L.D., C.F., A.D.I., G.H.K., S.M.L., Y.‐A.L., L.P., N.J.R., C.S., and M.S. drafted the work or substantively revised it. All authors have approved the submitted version.

## Conflicts of Interest

S.J.B. has received research funding (but no personal financial benefits) from the Wellcome Trust (220875/Z/20/Z), UKRI, Medical Research Council, Rosetrees Trust, Stoneygates Trust, British Skin Foundation, Charles Wolfson Charitable Trust, anonymous donations from people with eczema, Unilever, Pfizer, Abbvie, Sosei‐Heptares, Janssen, and European Lead Factory (which includes multiple industry partners). S.J.B., A.B.‐A., K.B., A.D.I., G.H.K., C.S., S.M.L., and L.P. have received funding from the BIOMAP‐IMI consortium (EU H2020 project ref. no. 821511) which receives support from several pharmaceutical industry partners. L.P. has received an honorarium payment for a scientific talk on eczema genetics from LEO Pharma. G.H.K. reports grants from the Netherlands Lung Foundation, ZON‐MW, Ubbo Emmius Foundation, TEVA the Netherlands, GSK, Vertex, outside the submitted work (money to institution). His institution received compensation for consultancy or lectures from Astra Zeneca, Boehringer Ingelheim, and Sanofi.

## Supporting information


**File S1.** Table of genetic variants, risk alleles and risk allele frequencies.
**File S2.** Cohort descriptions.
**File S3.** Definition and coding of environmental exposures.
**File S4.** List of cohorts and available exposure data.
**File S5.** Logistic regression models on ever having atopic eczema.
**File S6.** Estimation of statistical power. Posthoc power calculations performed to facilitate interpretation of negative findings.
**File S7.** Human keratinocyte culture and in vitro analysis for rs10214237 × dog interaction.
**File S8.** Full results of the discovery analysis.
**File S9.** Full results of the replication analysis.
**File S10.** *IL7R* mRNA expression in cells of different rs10214237 genotype.
**File S11.** Results of cytokine, chemokine and receptor expression on human primary keratinocytes following dog allergen exposure.

## Data Availability

The data that support the findings of this study are available on request from the corresponding author. The data are not publicly available due to privacy or ethical restrictions.
